# Specific and sensitive GC–MS analysis of hypusine, *N*^ε^-(4-amino-2-hydroxybutyl)lysine, a biomarker of hypusinated eukaryotic initiation factor eIF5A, and its application to the bi-ethnic ASOS study

**DOI:** 10.1007/s00726-022-03142-8

**Published:** 2022-03-03

**Authors:** Svetlana Baskal, Annette Kaiser, Catharina Mels, Ruan Kruger, Dimitrios Tsikas

**Affiliations:** 1 Core Unit Proteomics, Institute of Toxicology, Hannover Medical School, Carl-Neuberg-Strasse 1, 30625 Hannover, Germany; 2 Medical Research Centre, University Hospital, University Duisburg-Essen, Hufelandstrasse 55, 45128 Essen, Germany; 3 Hypertension in Africa Research Team (HART), North-West University, Potchefstroom, South Africa; 4 MRC Research Unit for Hypertension and Cardiovascular Disease, North-West University, Potchefstroom, South Africa

**Keywords:** AGEs, Amino acids, ASOS, Derivatization, Deuterium, eIF5A, GC–MS, Hypusine, Post-translational modification (PTM), Quantification

## Abstract

Hypusination is a unique two-step enzymatic post-translational modification of the *N*^ε^-amino group of lysine-50 of the eukaryotic initiation factor 5A (eIF5A). We developed a specific and sensitive gas chromatography–mass spectrometry (GC–MS) method for the measurement of biological hypusine (Hyp), i.e., *N*^ε^-(4-amino-2-hydroxybutyl)lysine. The method includes a two-step derivatization of Hyp: first esterification with 2 M HCl in CH_3_OH (60 min, 80 °C) to the methyl ester (Me) and then acylation with penta-fluoro-propionic (PFP) anhydride in ethyl acetate (30 min, 65 °C). Esterification with 2 M HCl in CD_3_OD was used to prepare the internal standard. The major derivatization product was identified as the un-labelled (d_0_Me) and the deuterium-labelled methyl esters (d_3_Me) derivatives: d_0_Me-Hyp-(PFP)_5_ and d_3_Me-Hyp-(PFP)_5_, respectively. Negative-ion chemical ionization generated the most intense ions with *m*/*z* 811 for d_0_Me-Hyp-(PFP)_5_ and *m*/*z* 814 for the internal standard d_3_Me-Hyp-(PFP)_5_. Selected-ion monitoring of *m*/*z* 811 and *m*/*z* 814 was used in quantitative analyses. Free Hyp was found in spot urine samples (10 µL) of two healthy subjects at 0.60 µM (0.29 µmol Hyp/mmol creatinine) in the female and 1.80 µM (0.19 µmol Hyp/mmol creatinine) in the male subject. The mean accuracy of the method in these urine samples spiked with 1–5 µM Hyp was 91–94%. The limit of detection (LOD) of the method is 1.4 fmol Hyp. The method was applied to measure the urinary excretion rates of Hyp in healthy black (*n* = 38, age 7.8 ± 0.7 years) and white (*n* = 41, age 7.7 ± 1.0 years) boys of the Arterial Stiffness in Offspring Study (ASOS). The Hyp concentrations were 3.55 [2.68–5.31] µM (range 0.54–9.84 µM) in the black boys and 3.87 [2.95–5.06] µM (range 1.0–11.7 µM) in the white boys (*P* = 0.64). The creatinine-corrected excretion rates were 0.25 [0.20–0.29] µmol/mmol (range 0.11–0.36 µmol/mmol) in the black boys and 0.26 [0.21–0.30] µmol/mmol (range 0.10–0.45 µmol/mmol) in the white boys (*P* = 0.82). These results suggest that there is no ethnic-related difference in the ASOS population in the eIF5A modification. Remarkable differences were found between black and white boys with respect to correlations of urinary Hyp with amino acids and advanced glycation end-products of Lys, Arg and Cys. Deoxyhypusine, formally the direct precursor of Hyp, seems not to be excreted in the urine by healthy subjects.

## Introduction

**Scheme 1:**
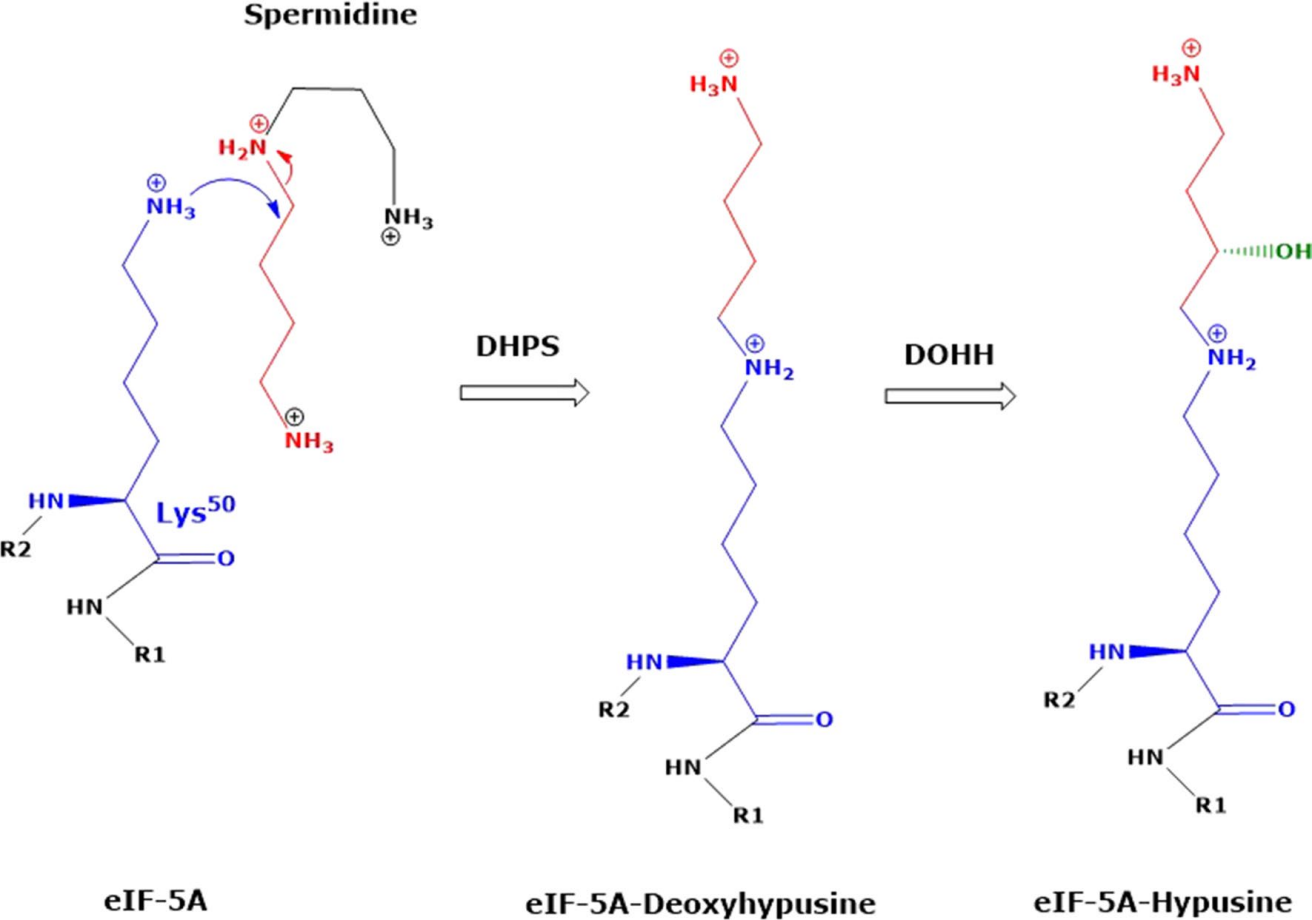
Simplified schematic of the enzymatic formation of hypusine from the eukaryotic initiation factor 5A (eIF5A). Deoxy-hypusine synthase (DHPS) catalyzes the cleavage of the 4-aminobutyl moiety of spermidine and the transfer to the ε-amino group of the lysine residue number 50 (K-50) of eIF5A to form deoxyhypusinated eIF5A. This is subsequently hydroxylated by deoxyhypusine hydroxylase (DOHH) to form hypusinated eIF5A. In vivo, proteolysis of hypusinated eIF5A releases hypusine, i.e., *N*^ε^-(4-amino-2-hydroxybutyl)lysine (not shown)

Residues of proteinogenic amino acids undergo multiple chemical and enzymatic post-translational modifications (PTM). Hypusination is a unique two-step enzymatic PTM (Scheme 1). It occurs exclusively on the *N*^ε^-amino group of a single lysine (Lys) residue, i.e., Lys^50^, in the eukaryotic initiation factor 5A (eIF5A). eIF5A promotes translation, elongation and facilitates translation termination. Hypusination is required for the activity of eIF5A, mammalian cell proliferation, and neurodevelopment. eIF5A has been implicated in various human pathological conditions including infectious diseases, diabetes, neurodevelopment disorders and cancer (Nakanishi and Cleveland 2016; McNamara et al. 2021; Park et al. 2021). The two-step biosynthesis of hypusine is illustrated in Scheme 1. Deoxyhypusine synthase (DHPS, EC 2.5.1.46) catalyzes the cleavage of the 4-aminobutyl moiety of the polyamine spermidine and its transfer to the ε-amino group of the Lys^50^ residue of eIF5A to form deoxyhypusinated eIF5A (eIF5A^DHP^), which is subsequently hydroxylated by deoxyhypusine hydroxylase (DOHH, EC 1.14.99.29) to form the hypusinated eIF5A (eIF5A^HYP^). In in vivo, proteolysis of eIF5A^HYP^ releases hypusine (Hyp), i.e., *N*^ε^-(4-amino-2-hydroxybutyl)lysine. In in vitro, Hyp can be released from eIF5A^HYP^ by classical HCl-catalyzed hydrolysis.

### Brief retrospect of hypusine analysis

Hyp was first identified in a protein of human lymphocytes with spermidine being the biosynthetic precursor (Park et al. 1981). The first report on the occurrence and quantity of free Hyp in mammalian organs, human urine and human serum has been reported in 1971 (Nakajima et al. 1971). Healthy humans were found to excrete about 110 nmol Hyp per mmol creatinine (corresponding to concentrations of 1–5 µM in the urine); the serum Hyp concentration was reported to be below 200 nM (Nakajima et al. 1971). Children with hyperlysinemia were found to excrete 7–10 times higher amounts of free Hyp compared to controls (730–1934 vs. 37–271 nmol Hyp per 24 h), but to have serum concentrations of hypusine of about 200 nM, i.e., comparable to those of healthy controls (Woody and Pupene 1973). In the above-mentioned publications, Hyp has been analyzed by commercially available automatic amino acid analyzers without reporting the analytical performance of this technique (Nakajima et al. 1971; Woody and Pupene 1973). These studies did not report on the occurrence of deoxy-hypusine (*N*^ε^-(4-amino-butyl)lysine).

Later, high-performance liquid chromatography (HPLC) with *o*-phthaldialdehyde and dansyl derivatization was used to identify a Hyp-containing dipeptide, i.e., α-(β-alanine)hypusine, in bovine brain (Ueno et al. 1991). HPLC with dansyl derivatization was used to analyze Hyp and deoxyhypusine (dHyp) from eIF5A rice plants (Mehta et al. 1994). Dansyl-hypusine in collected HPLC fractions was esterified and analyzed by gas chromatography–mass spectrometry (GC–MS) in in vitro investigations (Mehta et al. 1994). This technique was used in in vitro investigations of T cells (Bergeron et al. 1998). More recently, an ultra-performance liquid chromatography (UPLC) method with *o*-phthaldialdehyde derivatization was found useful as an assay for determining DHPS and DOHH activity (measurement of Hyp and dHyp) and testing the inhibition of novel of antimalarials (Kaiser et al. 2012).

GC–MS is a useful technique for the quantitative analysis of amino acids and their metabolites after chemical derivatization (Hušek et al. 2016). One of our groups analyzed biological Hyp and dHyp by GC–MS (Frommholz et al. 2009; Atemnkeng et al. 2013; von Koschitzky and Kaiser 2013; von Koschitzky et al. 2015). Biological Hyp and dHyp were identified in proteolysates by GC–MS after derivatization with chloroformate (Frommholz et al. 2009; Atemnkeng et al. 2013). Biological Hyp and dHyp were also analyzed by GC–MS using a three-step derivatization procedure and electron ionization (EI) (von Koschitzky et al. 2015). This procedure included esterification with ethanol/acetyl chloride to prepare the ethyl ester of Hyp and dHyp, *N*-acylation with tri-fluoro-acetic anhydride/tri-fluoro-acetic acid ethyl ester to prepare the amides of Hyp and deoxy-hypusine, and silylation with hexamethyldisilazane to trimethylsilylate the hydroxy group of Hyp (von Koschitzky et al. 2015).

Recently, we have reported on the quantitative analysis of native and *N*^ε^-methylated and *N*^ε^-glycated Lys in biological samples including human urine by GC–MS (Baskal et al. 2021). Generally, we use negative-ion chemical ionization (NICI), because this ionization technique provides considerably higher sensitivity than EI. This is due the “softer” ionization resulting from the high electronegativity of fluorine (F) in combination with the use of a buffer gas (i.e., methane). As endogenous substances do not contain F atoms in their molecules, they are introduced using perfluorated derivatization reagents, such as pentafluoropropionic anhydride (PFPA) (Tsikas et al. 2003) and pentafluorobenzyl bromide (PFB-Br) (Tsikas 2017a).

In the present work, we tested the utility of PFPA for the derivatization of Hyp and its analysis by GC–MS in the NICI mode. Previously, we found that PFPA derivatization is useful for the quantitative measurement of 5-hydroxy-lysine in biological samples (Baskal et al. 2021). We found that 5-hydroxy-lysine, which is structurally related to Hyp, does not need a separate derivatization step for its hydroxyl group, because it is also acylated (Baskal et al. 2021). Thus, both aliphatic hydroxy and amine groups of amino acids and their metabolites can be simultaneously derivatized by PFPA. Waiving a separate derivatization step of the hydroxy group of Hyp would simplify and shorten considerably the analytical process. In situ preparation of tri-deutero-methyl esters (d_3_Me) of amino acids is a convenient laboratory method to generate stable-isotope labelled analogs for use as internal standards in quantitative analyses (Tsikas 2009a). This technique overcomes problems arising from the lack of commercially available stable-isotope labelled reference compounds.

The bi-ethnic Arterial Stiffness in Offspring study (ASOS) was originally conducted to investigate the link of urinary metabolites with premature arterial stiffness and the early detection and identification of cardiovascular disease and hypertension development in black and white populations from South Africa (Erasmus et al. 2019). This study revealed an association of pulse wave velocity with proline, a precursor of spermidine, which, in turn, is a precursor of Hyp. It was, therefore, reasonably to apply the GC–MS method to the ASOS study to investigate potential ethnic-associated differences with respect to eIF5A. In a previous study, we did not find ethnic-dependent differences in healthy black and white boys with respect to Lys and its advanced glycation end-products (AGEs) (Baskal et al. 2021).

## Materials and methods

### Chemicals, materials and reagents

The (2*S*)-Hypusine dihydrochloride (declared chemical purity, ≥ 95% by HPLC), tetradeutero-methanol (CD_3_OD; declared isotopic purity, ≥ 99.8% at ^2^H) and penta-fluoro-propionic anhydride (PFPA) were purchased from Sigma-Aldrich (Steinheim, Germany). Methanol (CH_3_OH) was obtained from Chemsolute (Renningen, Germany). Hydrochloric acid (37 wt%) was purchased from Baker (Deventer, The Netherlands). Ethyl acetate (EA) was obtained from Merck (Darmstadt, Germany). Glassware for GC–MS (1.5-mL auto-sampler vials and 0.2-mL micro-vials) and the fused-silica capillary column Optima 17 (15 m × 0.25 mm I.D., 0.25-µm film thickness) were purchased from Macherey–Nagel (Düren, Germany). Stock solutions of Hyp were prepared by dissolving the commercial preparation in its original glass flask in deionized water. The stock solution of Hyp was diluted with deionized water as appropriate.

### Derivatization procedures for hypusine

For the preparation of un-labelled methyl esters and deuterium-labelled methyl esters of Hyp, i.e., d_0_Me-Hyp and d_3_Me-Hyp, respectively, two derivatization reagents were used. The esterification reagent 2 M HCl/CH_3_OH was prepared by slowly adding, under gentle mixing, 16 mL of 37 wt% HCl to 80 mL ice-cold CH_3_OH. Analogous, to 80 mL of ice-cold CD_3_OD was added 16 mL of 37 wt% HCl slowly, under gentle mixing, to obtain the esterification reagent 2 M HCl/CD_3_OD. The concentration of HCl in these methanolic solutions was each 2 M. The acylation reagent PFPA in EA (PFPA/EA) was prepared daily by diluting pure PFPA in EA (1:4, v/v).

Methyl esters of Hyp were prepared as follows. Aqueous solutions of Hyp (0–10 µL, 10 mM) were evaporated to dryness under a stream of nitrogen. Then, the solid residues were reconstituted in 100-µL aliquots of 2 M HCl/CH_3_OH or 2 M HCl/CD_3_OD solutions and the vials were tightly sealed. Esterification was performed separately by heating the samples for 60 min at 80 °C. After cooling to room temperature, the solvents of the samples containing d_0_Me-Hyp and d_3_Me-Hyp were evaporated to dryness under a stream of nitrogen. Subsequently, 100-µL aliquots of the PFPA/EA were added, the glass vials were tightly sealed and heated for 30 min at 65 °C to prepare penta-fluoro-propionic (PFP) derivatives of the methyl esters. After cooling to room temperature, solvents and reagents were evaporated to dryness under a stream of nitrogen. Subsequently, the solid residues were treated first with 200-µL aliquots of 400 mM borate buffer, pH 8.5, and immediately thereafter with 200-µL aliquots of toluene, followed by immediate vortex-mixing for 60 s and centrifugation (4000×*g*, 5 min, 18 °C). This step was used to eliminate potential acidic components from hydrolyzed and reacted PFPA such as penta-fluoro-propionic acid and to extract the methyl ester pentafluoropropionyl (Me-PFP) derivatives into toluene. Aliquots (150 µL) of the upper organic phase were transferred into micro-inserts placed in auto-sampler glass vials, the vials were sealed, and the samples were subjected to GC–MS analysis.

### GC–MS conditions

All analyses were performed on a GC–MS apparatus consisting of a single-stage quadrupole mass spectrometer model ISQ, a Trace 1210 series gas chromatograph and an AS1310 autosampler from ThermoFisher (Dreieich, Germany). Toluene extracts (1 µL) were injected in the split-less mode. A 10-µL Hamilton needle of the auto-sampler was cleaned automatically three times with toluene (5 µL) after each injection. Injector temperature was kept at 280 °C. Helium was used as the carrier gas at a constant flow rate of 1.0 mL/min. Two oven temperature programs were applied. GC program #1: the oven temperature was held at 40 °C for 0.5 min and ramped to 320 °C at a rate of 15 °C/min and was held at this temperature for 1 min. GC program #1 was used in qualitative analyses. GC program #2: the oven temperature was held at 40 °C for 0.5 min and ramped first to 160 °C at a rate of 30 °C/min and then to 320 °C at a rate of 15 °C/min and was held at this temperature for 1 min. GC program #2 was used in quantitative analyses. Interface and ion-source temperatures were set to 300 °C and 250 °C, respectively. Electron energy was 70 eV and electron current 50 µA. Methane was used as the buffer (reactant) gas for NICI at a constant flow rate of 2.4 mL/min. The electron multiplier voltage was routinely set to 1400 V.

### Subjects: the arterial stiffness in offspring study (ASOS)

We applied the current method to the quantification of Hyp in 10-µL aliquots from spot urine samples of 38 healthy black boys (age 7.8 ± 0.7 years) and 41 healthy white boys (age 7.7 ± 1.0 years) collected in a previous study after approval by the local Ethics Committee (Mokwatsi et al. 2017). Ethical statement: participants were fully informed about the objective of the study (written informed consent and assent were obtained from all participants included in the study). All procedures performed in the study were in accordance with the ethical standards of the institutional and/or national research committee (Health Research Ethics Committee of the North-West University; NWU-00007-15-A1) and with the 1964 Helsinki Declaration and its later amendments or comparable ethical standards (Carlson et al. 2004).

### Statistical analyses

GraphPad Prism 7 for Windows (GraphPad Software, San Diego, CA, USA) was used to test linearity, normality, statistical significance using two-tailed unpaired *t* test and to prepare graphs.

## Results

### GC–MS characterization of hypusine derivatives

Mass spectra of Hyp were obtained after derivatization of two aliquots containing each 0.5 µmol of the synthetic amino acid Hyp with 2 M HCl/CH_3_OH and 2 M HCl/CD_3_OD, respectively, followed by derivatization with PFPA/EA as described above. Derivatives were extracted with toluene (200 µL), and 1-µL aliquots containing each 2.5 nmol of the derivatized analyte (assuming quantitative yield) were injected in the GC–MS apparatus in the split-less mode. NICI mass spectra of Hyp derivatives were generated by scanning the quadrupole in the *m*/*z* range 100–1000 with a scan rate of 1 s per cycle. The structures of the derivatives and the mass fragments were elucidated considering the expected 3-Da difference between un-labelled and deuterium-labelled analytes and the expected shorter retention times of derivatives containing chemically introduced deuterium atoms (Baskal et al. 2021).

**Fig. 1:**
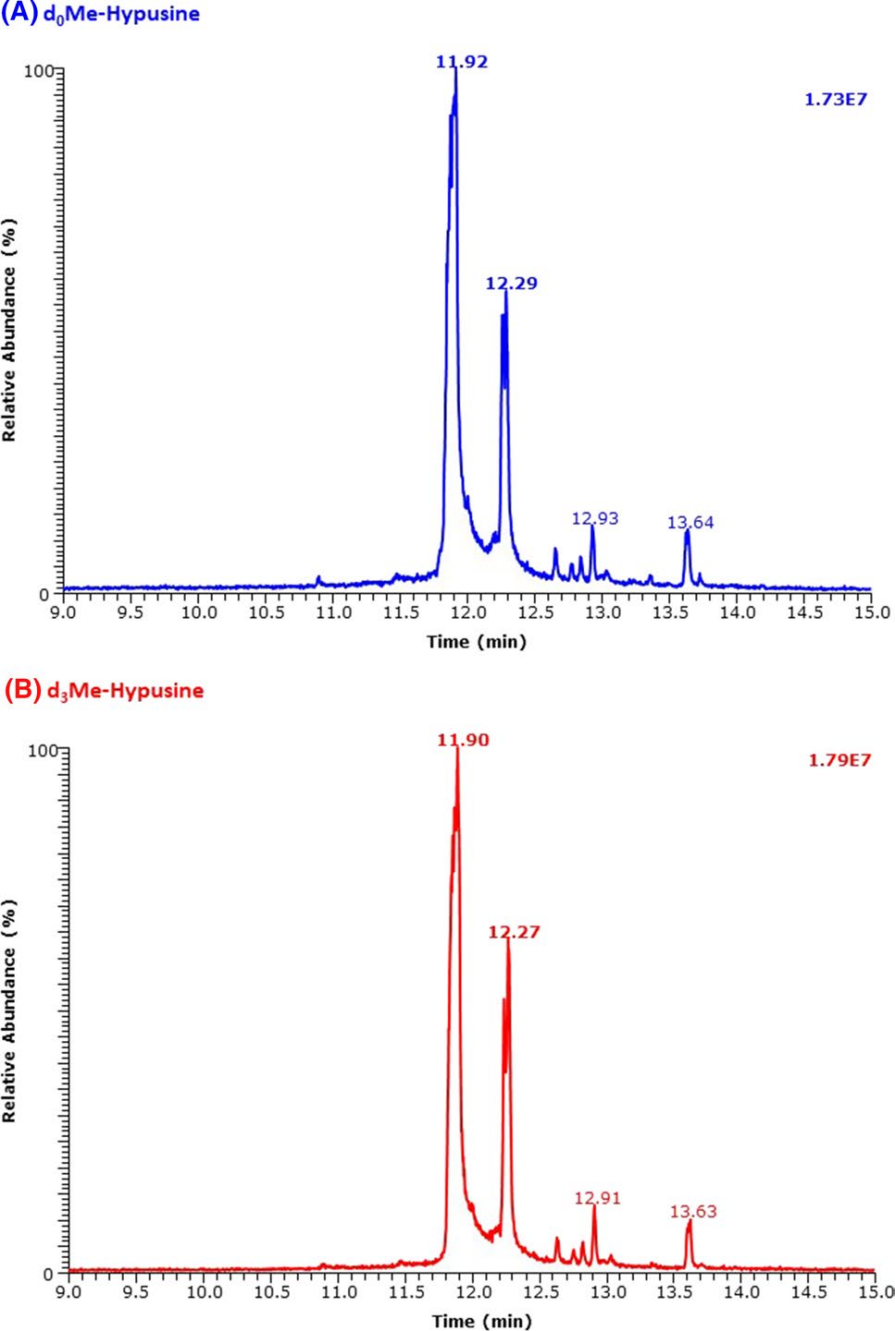
GC–MS chromatograms obtained from the analysis each of 2.5 nmol (injected) of the methyl ester penta-fluoro-propionic derivatives **A** of the un-labelled hypusine (d_0_Me-hypusine) and **B** of the deuterium-labelled hypusine (d_3_Me-hypusine). Negative-ion chemical ionization and scanning in the *m*/*z* range 100–1000 (1 s per cycle) were performed. The GC oven temperature program #1 was used

The two-step derivatization of synthetic Hyp resulted in two major and two minor GC peaks using the gentle GC oven temperature program #1 (Fig. 1). All GC peaks of the trideuteromethyl esters eluted each in front of the corresponding peaks of the un-labelled methyl esters, indicating the presence of deuterium atoms in the derivatives prepared using 2 M HCl/CD_3_OD.

**Fig. 2:**
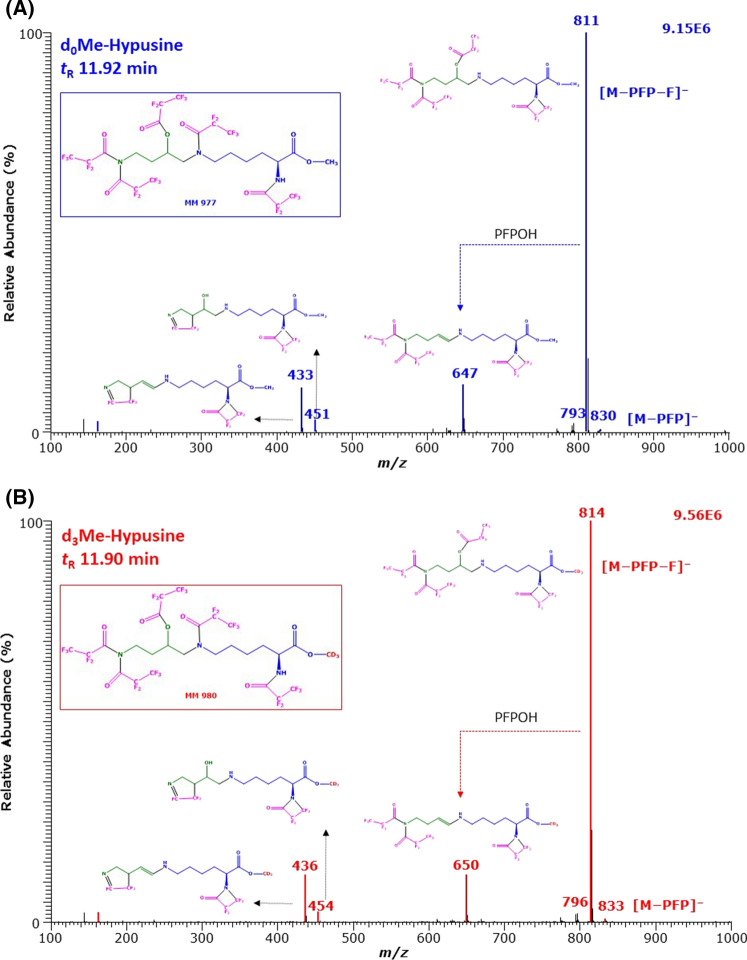
GC–MS spectra of the methyl ester penta-fluoro-propionyl derivatives of **A** the un-labelled (d_0_) and **B** the deuterium-labelled (d_3_) hypusine (*N*^ε^-(4-amino-2-hydroxybutyl)lysine) eluting at 11.92 min and 11.90 min. Negative-ion chemical ionization and scanning in the *m*/*z* range 100–1000 (1 s per cycle) were performed. The injected amount was each 2.50 nmol hypusine. The GC oven temperature program #1 was used. Inserts show the proposed structures of the mass fragments. *Me* methyl ester, *PFP* pentafluoropropionyl, *PFPOH* pentafluoropropionic acid, *MM* molecular mass. The proposed structure of the derivatives is framed. See also Fig. 1

The NICI mass spectra of the GC–MS peaks with the retention times of 11.92 min and 11.90 min are shown in Fig. 2. Almost all corresponding mass fragments differed by 3 Da, strongly indicating the presence of the trideuteromethyl (d_3_Me) group in the carboxylic group of these anions. The most intense ions (base peaks; intensity, 100%) with *m*/*z* 811 and *m*/*z* 814 are most likely due to [M–PFP–F]^−^. The less intense ions (intensity, 10%) with *m*/*z* 647 and *m*/*z* 650 are presumably due to [M–PFP–F–PFPOH]^−^ (Fig. 2). The very weak complementary ions *m*/*z* 830 and *m*/*z* 833 are likely to result from neutral loss of PFP from the derivative (i.e., [M–PFP]^−^). The mass spectra of the major Hyp derivatives suggest that all functional groups of the Hyp methyl esters including the hydroxyl group of Hyp are derivatized with PFPA. Likely structures for the Hyp derivatives eluting at 11.92 min and 11.90 min could be due to Hyp-d_0_Me-(*N,N,N,N,O*-PFP)_5_ for the un-labelled Hyp and Hyp-d_3_Me-(*N,N,N,N,O*-PFP)_5_ for the labelled Hyp derivative.

**Fig. 3:**
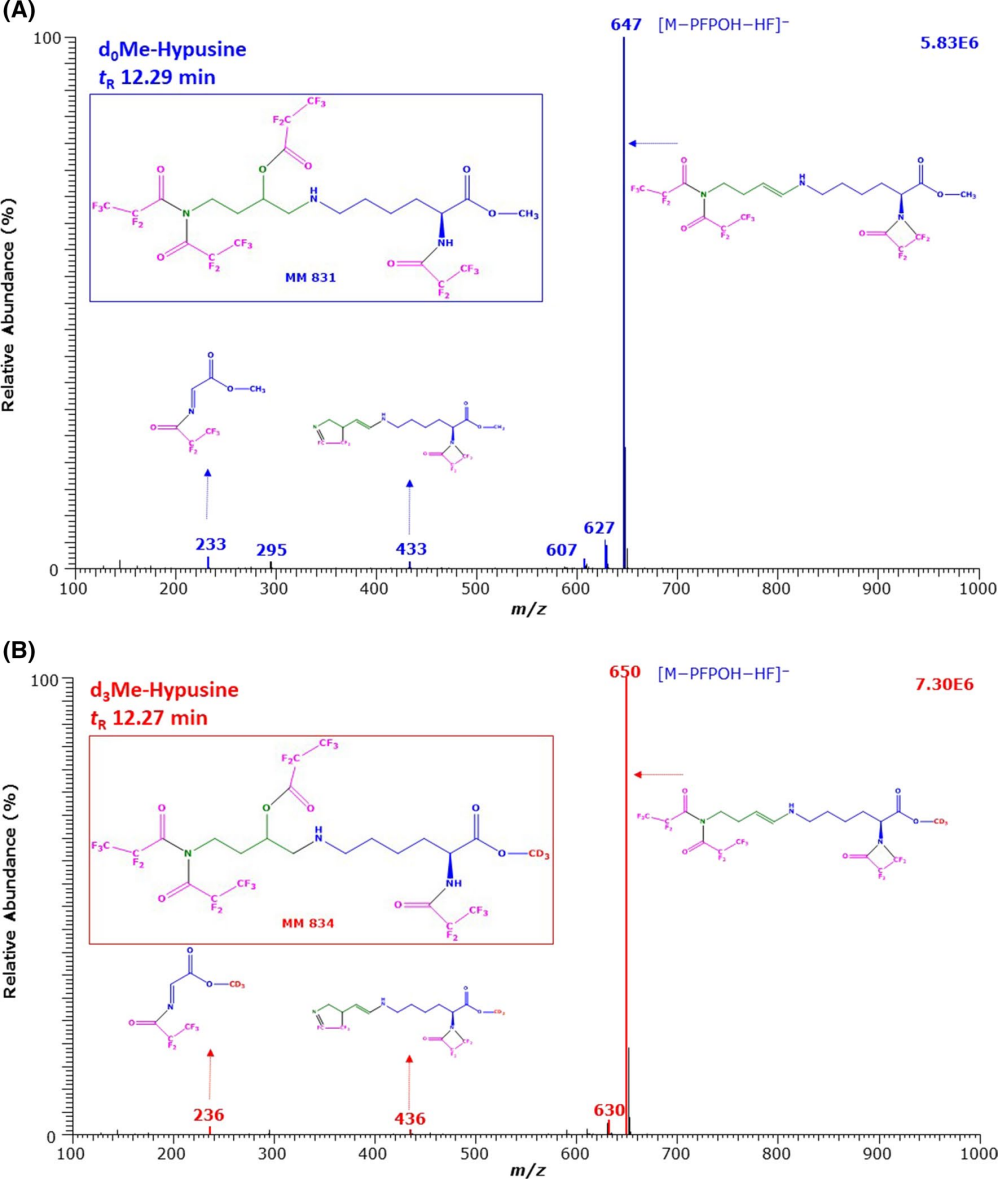
GC–MS spectra of the methyl ester pentafluoropropionyl derivatives of (upper panel) the unlabelled and (lower panel) the deuterium-labelled hypusine (*N*^ε^-(4-amino-2-hydroxybutyl)lysine) eluting at 12.29 min and 12.27 min. The GC oven temperature program #1 was used. Negative-ion chemical ionization and scanning in the *m*/*z* range 100–1000 (1 s per cycle) were performed. The injected amount was each 2.5 nmol hypusine. Inserts show the proposed structures of the mass fragments. *Me* methyl ester, *PFP* pentafluoropropionyl, *PFPOH* pentafluoropropionic acid, *MM* molecular mass. The proposed structure of the derivatives is framed. See also Figs. 1 and 2

The mass spectra of the second large GC–MS peaks with the retention times of 12.29 min and 12.27 min are shown in Fig. 3. Again, almost all corresponding (complementary) mass fragments differed by 3 Da, strongly indicating the presence of the methyl ester in these anions. The most intense ions (base peaks; intensity, 100%) with *m*/*z* 647 and *m*/*z* 650 are most likely due to [M–PFPOH–HF]^−^. The ions with *m*/*z* 433 and *m*/*z* 436 were also obtained, but they are of much less intensity (relative abundance, 2%) compared to that of the first GC–MS peak (Fig. 2). The longer retention time of the second major Hyp derivatives in combination with the mass spectra suggests that the hydroxyl groups, but not the *N*^ε^-amine groups, of these Hyp derivatives are PFP acylated. It seems that under the GC–MS conditions, the acylated hydroxy groups of these Hyp derivatives leave the molecules to generate each one olefinic group, analogous to d,l-5-hydroxy-l-lysine (Baskal et al. 2021). Interestingly, the ions with *m*/*z* 647 and *m*/*z* 650 of the second GC–MS peaks (Fig. 3) are also observed in the earlier eluting GC–MS peaks (Fig. 2), but are of considerably lower intensity. Likely structures for the Hyp derivatives eluting at 12.2 min could be Hyp-d_0_Me-(*N,N,N,O*-PFP)_4_ for un-labelled Hyp and Hyp-d_3_Me-(*N,N,N,O*-PFP)_4_ for the deuterium-labelled Hyp derivative.

**Fig. 4:**
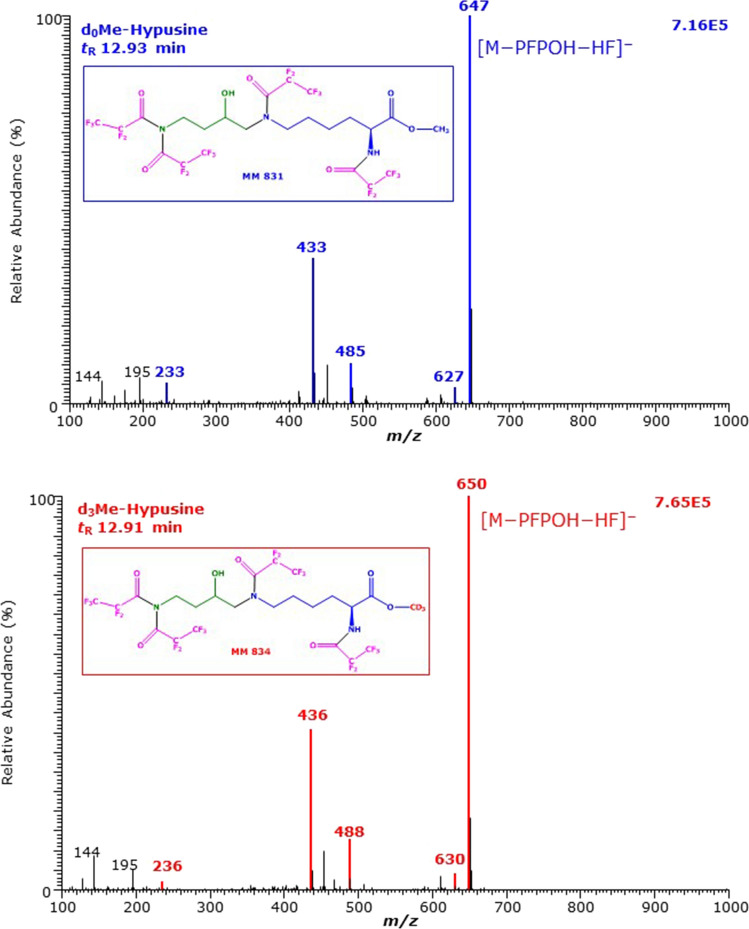
GC–MS spectra of the methyl ester pentafluoropropionyl derivatives of (upper panel) the unlabelled and (lower panel) the deuterium-labelled hypusine (*N*^ε^-(4-amino-2-hydroxybutyl)lysine) eluting at 12.93 min and 12.91 min. The GC oven temperature program #1 was used. Negative-ion chemical ionization and scanning in the *m*/*z* range 100–1000 (1 s per cycle) were performed. The injected amount was each 2.5 nmol hypusine. Inserts show the proposed structures of the mass fragments. *Me* methyl ester, *PFP* pentafluoropropionyl, *PFPOH* pentafluoropropionic acid, *MM* molecular mass. The proposed structure of the derivatives is framed. See also Figs. 1, 2 and 3

Analogous considerations suggest that the smaller GC–MS peaks eluting at 12.93 min and 12.91 min are due to Hyp-d_0_Me-(*N,N,N,N*-PFP)_4_ and Hyp-d_3_Me-(*N,N,N,N*-PFP)_4_ and they contain a non-acylated hydroxyl group (Fig. 4). This hydroxyl group seems to leave the derivatives as water and to generate the moderately intense ions with *m*/*z* 433 and *m*/*z* 436, respectively, and to prolong the retention time relative to the GC–MS peaks eluting at 12.29 and 12.27 min.

**Fig. 5:**
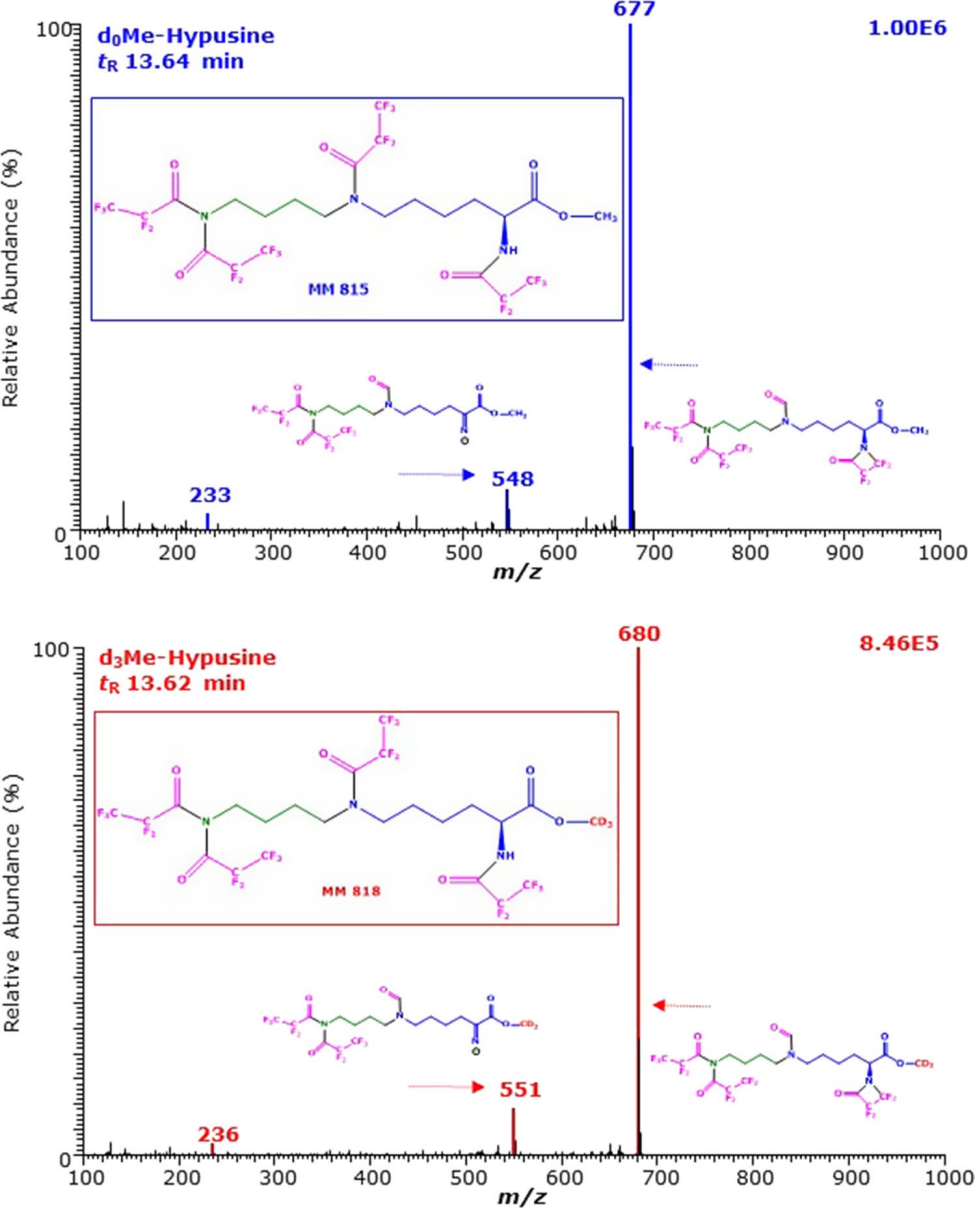
GC–MS spectra of the methyl ester pentafluoropropionyl derivatives of (upper panel) the unlabelled and (lower panel) the deuterium-labelled hypusine (*N*^ε^-(4-amino-2-hydroxybutyl)lysine) eluting at 13.64 min and 13.62 min. The GC oven temperature program #1 was used. Negative-ion chemical ionization and scanning in the *m*/*z* range 100–1000 (1 s per cycle) were performed. The injected amount was each 2.5 nmol hypusine. Inserts show the proposed structures of the mass fragments. *Me* methyl ester, *MM* molecular mass. The proposed structure of the derivatives is framed. See also Figs. 1, 2, 3 and 4

The lastly eluting small GC–MS peaks at 13.64 min and 13.62 min (Fig. 1) have the most intense ions at *m*/*z* 677 and *m*/*z* 680 and weak ions at *m*/*z* 548 and *m*/*z* 551 (Fig. 5), indicative of their methyl ester groups. These ions have not been observed in the GC–MS spectra of the three other peaks discussed above. The parent compound of these derivatives could be deoxyhypusine (dHyp) which do not contain a hydroxyl group. Possible structures for these derivatives could be dHyp-d_0_Me-(*N,N,N,N*-PFP)_4_ and dHyp-d_3_Me-(*N,N,N,N*-PFP)_4_. The manufacturer declared a chemical purity of at least 95% without any information about the nature of potential impurities.

### GC–MS analysis of hypusine in aqueous samples

Freshly prepared solutions of synthetic Hyp in distilled water were used to test the linearity of the GC–MS method. For the internal standard, 10-µL aliquots of a 100-µM solution of Hyp corresponding to a total amount of 1000 pmol d_3_Me-Hyp were used. For Hyp, 0, 5, 10, 15 and 20 µL of the same 100-µM solution of synthetic Hyp were taken corresponding to amounts of 0, 500, 1000, 1500 and 2000 pmol. After evaporation of the solvents to dryness under a stream of nitrogen gas, separate esterification was performed. After cooling to temperature, the corresponding aliquots of un-labelled and deuterium-labelled samples were combined, and the solvents were evaporated to dryness under a stream of nitrogen gas. Then, acylation with PFPA/EA and extraction with toluene/borate buffer (200 µL/200 µL) was performed. Subsequently, the toluene phase (150 µL) was transferred into auto-sampler glass vials with micro-inserts and 1-µL aliquots thereof were subjected to GC–MS analysis. The rampant GC–MS oven temperature program #2 and selected ion monitoring (SIM) of *m*/*z* 811 and *m*/*z* 814 for the major peak were performed.

**Fig. 6:**
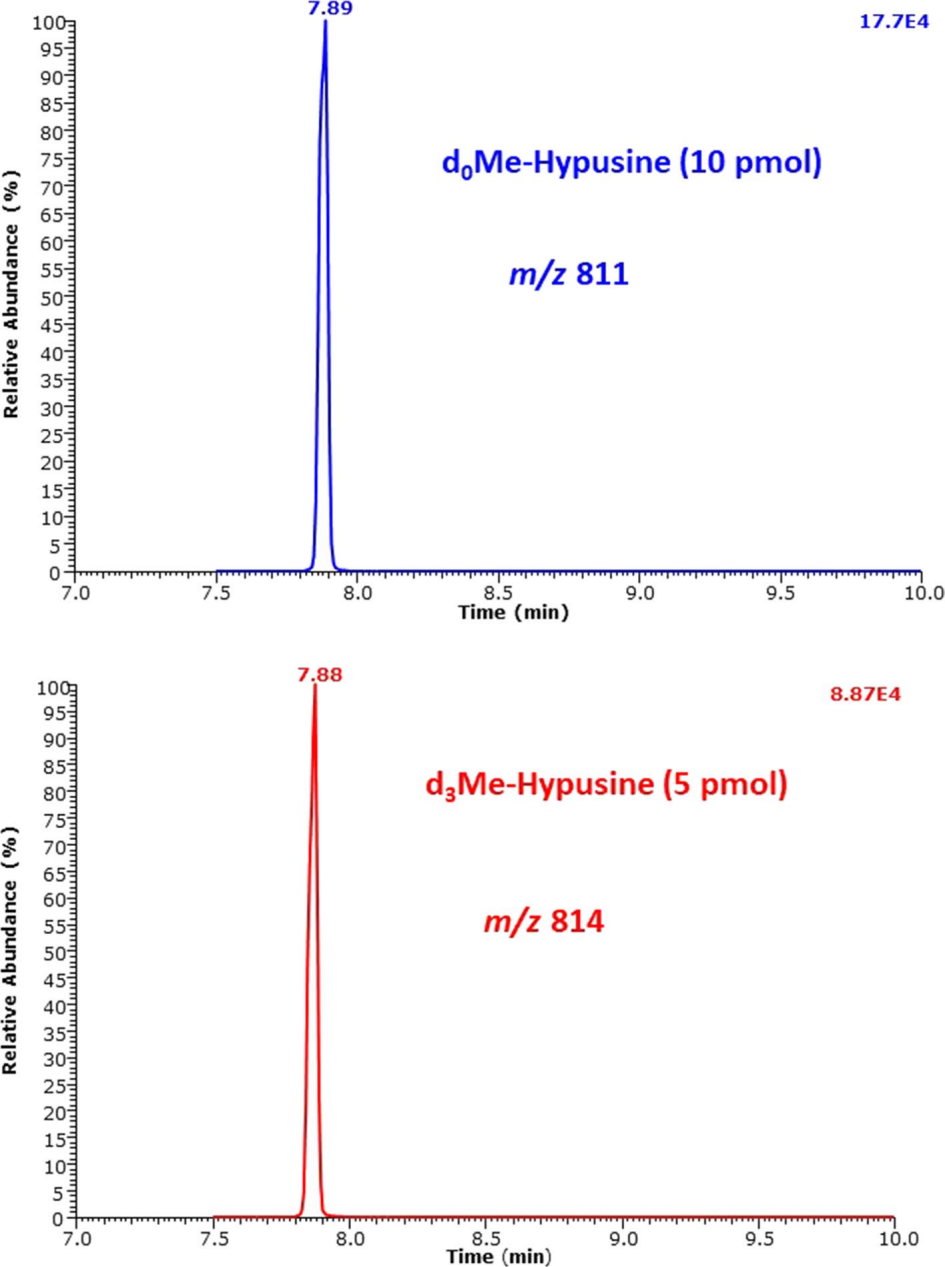
Partial chromatograms from the GC–MS analysis of synthetic hypusine (200 µM, 2000 pmol) using deuterium-labelled hypusine (100 µM, 1000 pmol) as the internal standard in distilled water. SIM of *m*/*z* 811 for d_0_-hypusine and *m*/*z* 814 for d_3_-hypusine in the negative-ion chemical ionization mode was performed. The GC oven temperature program #2 was used. The injected amounts were 10 pmol d_0_Me-hypusine and 5 pmol d_3_Me-hypusine

**Fig. 7:**
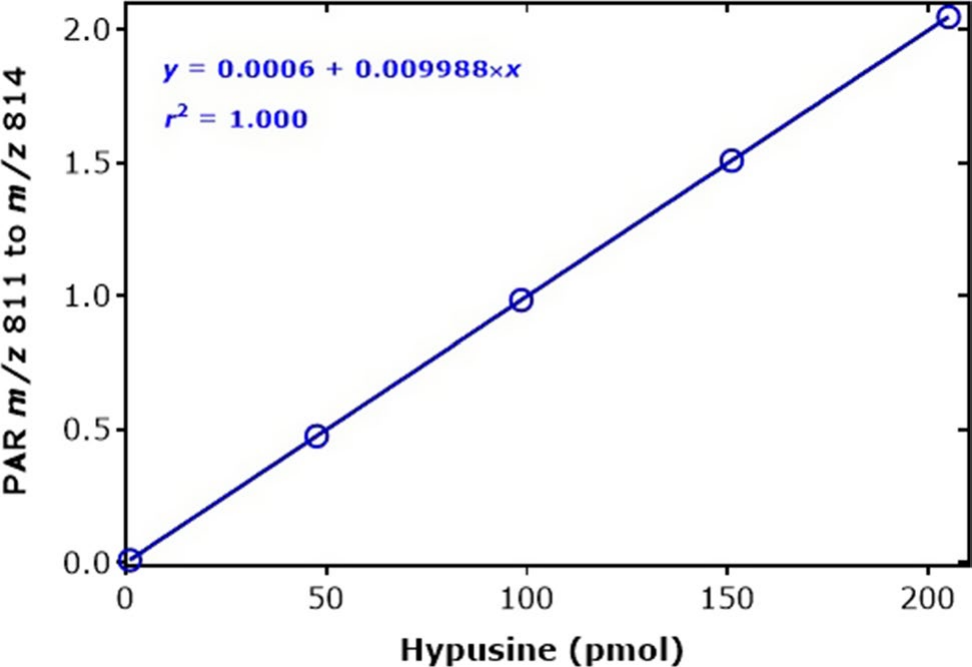
Peak area ratio (PAR) of *m*/*z* 811 to *m*/*z* 814 from the derivatization of the indicated amounts of hypusine and a fixed amount of 100 pmol of the internal standard in distilled water. SIM of *m*/*z* 811 for d_0_-hypusine and *m*/*z* 814 for d_3_-hypusine in the negative-ion chemical ionization mode was performed. The GC oven temperature program #2 was used. See also Fig. 6

Figure 6 shows a GC–MS chromatogram of a mixture of d_0_Me-Hyp and d_3_Me-Hyp after two-step derivatization. It contains a single peak for the d_0_Me-Hyp derivative and a single peak for the d_3_Me-Hyp derivative. Both peaks have a symmetric shape. The injected amounts were 10 pmol d_0_Me-Hyp and 5 pmol d_3_Me-Hyp. In this sample, the signal-to-noise ratio (*S*/*N*) was 22,219:1 for d_0_Me-Hyp and 10,696:1 for d_3_Me-Hyp. Considering that the limit of detection (LOD) is defined at an *S*/*N* of 3:1, these data suggest that the LOD of the method is 1.35 fmol d_0_Me-Hyp and 1.40 fmol d_3_Me-Hyp. Plotting the peak area ratio (PAR) of *m*/*z* 811 and *m*/*z* 814 (*y*) against the d_0_Me-Hyp amount (*x*) resulted in a straight line (*r*^2^ = 1.000) with the equation *y* = 0.0006 + 0.009988*x* (Fig. 7). The reciprocal slope value is 100.1 pmol and confirms the nominal d_3_Me-Hyp amount in the sample (i.e., 100.0 pmol). The accuracy of the method in terms of recovery was determined to range between 93 and 102% for the added Hyp amounts. Linearity (*r*^2^ = 0.9785) was also observed for the second major GC–MS peak by SIM of *m*/*z* 647 for d_0_Me-Hyp and *m*/*z* 650 for d_3_Me-Hyp, yet the peak area values were about 10 times smaller on a molecular basis (data not shown).

### Validation of the GC–MS method for hypusine in human urine

We performed analyses of Hyp in 10-µL aliquots of spot urine samples collected by two healthy young volunteers both of an age of 30 years. In a preliminary experiment, the spiked Hyp concentrations were supra-physiological 0, 50, 100, 150, 200, and 250 µM. The concentration of the internal standard was each 100 µM. After derivatization and extraction, 1-µL toluene extracts were injected and SIM of *m*/*z* 811 and *m*/*z* 814 was performed. Linear regression analysis between the PAR *m*/*z* 811 and *m*/*z* 814 (*y*) and the Hyp concentration (*x*) added to the urine samples resulted in straight lines in both urine samples. The regression equations were *y* = − 0.0038 + 0.0093*x* (*r*^2^ = 0.9987) in the urine of the female donor (volunteer #1) and *y* = − 0.0134 + 0.0081*x* (*r*^2^ = 0.9542) in the urine of the male donor (volunteer #2). The slope values indicate mean accuracy values of 93% and 81%, respectively. The *y*-axis intercepts suggest no presence of endogenous Hyp, yet this information is questionable because of the wide concentration range.

Reliable quantitative analysis in urine samples from healthy and sick human subjects is performed after successful method validation in relevant physiological/pathological concentration ranges (Tsikas 2009b). The same urine samples of the two above-mentioned volunteers were spiked with 0, 1, 2, 3, 4, and 5 µM of synthetic Hyp. These concentrations are considered relevant in human urine samples (Nakajima et al. 1971; Woody and Pupene 1973). The concentration of the internal standard was set to 2.0 µM to cover the entire concentration range.

**Fig. 8:**
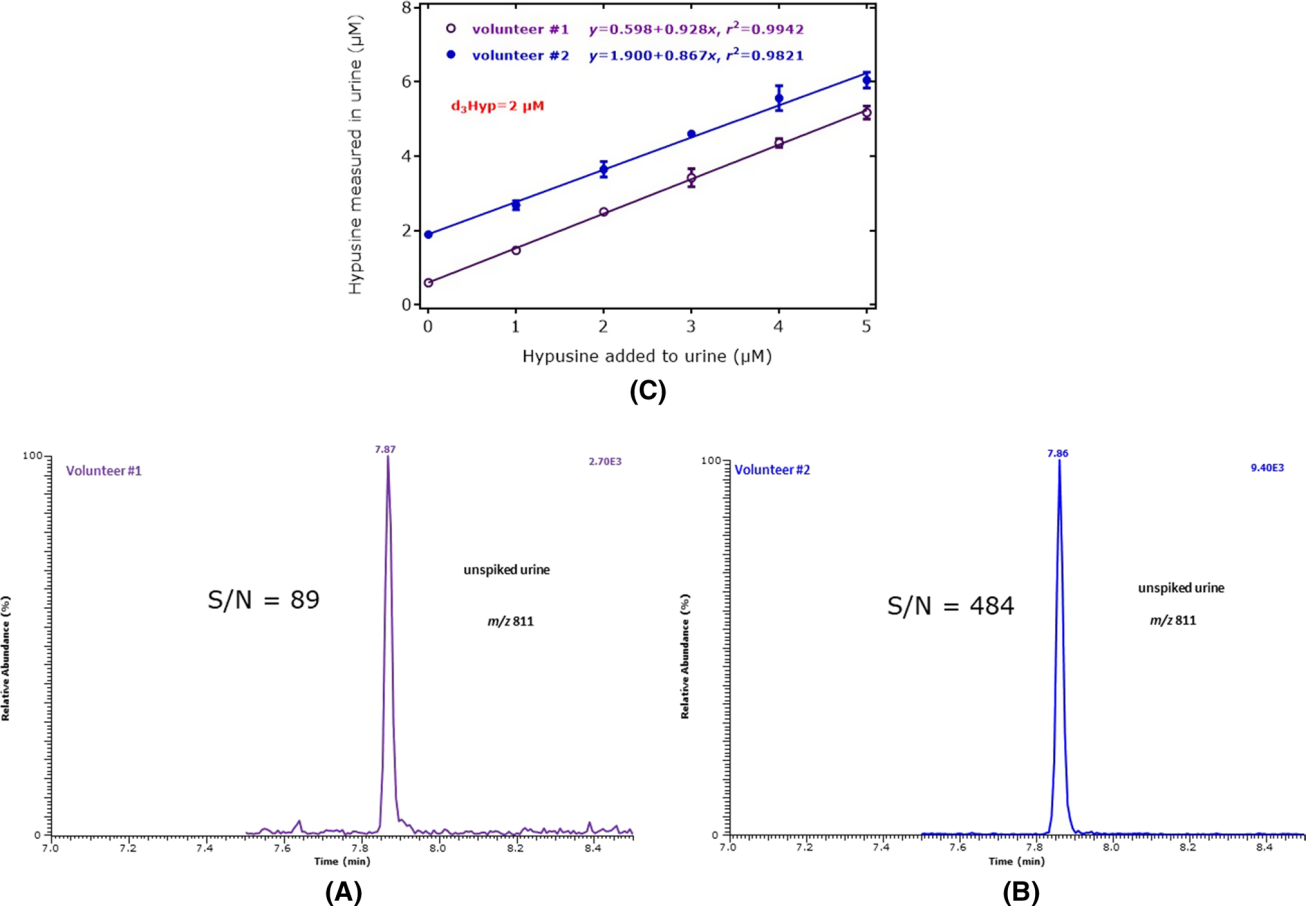
Partial chromatograms from the GC–MS analysis of hypusine in 10-µL aliquots of spot urine samples from volunteer #1 (**A**) and volunteer#2 (**B**) without addition of synthetic Hyp. SIM of *m*/*z* 811 for endogenous Hyp was performed. The GC oven temperature program #2 was used. **C** Relationship between the mean concentration of measured Hyp and the concentration of Hyp added to the urine samples #1 and #2 at the indicated levels. The error bars are standard deviations from triplicate analyse in both urine samples. The concentration of the internal standard d_3_MeHyp was each 2.0 µM in both urine samples. The creatinine concentration in the urine samples was 2.10 mM of the female and 9.66 mM of the male volunteer as measured by GC–MS (Tsikas et al. 2010). *S*/*N*, signal-to-noise ratio for endogenous hypusine in two human urine samples

The analytical performance of the GC–MS method in terms of accuracy (recovery) and the precision (relative standard deviation) in the validation experiment is summarized in Table 1. Figure 8 shows GC–MS chromatograms from the analysis of the urine samples of volunteers 1 and 2, to avoid potential contribution of synthetic Hyp to endogenous Hyp. In both urine samples, SIM of *m*/*z* 811 resulted each in a single GC–MS peak with the retention time of Hyp-d_0_Me-(*N,N,N,N,O*-PFP)_5_ using the GC oven program #2. Figure 8 also shows the plot of the Hyp concentration measured in the two urine samples (*y*) against the concentration of synthetic Hyp added to the urine samples (*x*). In the validation experiment, there were obtained straight lines with the regression equations *y* = 0.598 + 0.928*x* (*r*^2^ = 0.9942) in volunteer #1 and *y* = 1.90 + 0.867*x* (*r*^2^ = 0.9821) in volunteer #2. The *y*-intercept values indicate the presence of endogenous Hyp at basal concentrations of 0.60 µM in the urine of volunteer #1 and 1.90 µM in the urine of volunteer #2. Accuracy and precision of the GC–MS method for urinary Hyp lie in acceptable ranges. The creatinine-corrected concentration of endogenous Hyp corresponds to 0.286 nmol Hyp/mmol creatinine in volunteer #1 and 0.186 nmol Hyp/mmol creatinine in volunteer #2. The concentrations and the creatinine-corrected excretion rates of Hyp in the urine samples of the two volunteers lie in ranges reported previously using automated amino acid analyzers (Nakajima et al. 1971; Woody and Pupene 1973). They are of the same order of magnitude reported by us for Lys metabolites generated by other post-translational modifications using a similar GC–MS methodology (Baskal et al. 2021).

**Table 1:** Accuracy (recovery, %) and precision (RSD, %) of the GC–MS method for free hypusine in urine samples from two volunteers

Hyp added (µM)	Hyp measured (µM)	Recovery (%)	RSD (%)
Urine #1			
0	0.593 ± 0.011	Not applicable	1.95
1	1.463 ± 0.050	87.0	3.44
2	2.503 ± 0.050	95.5	2.01
3	3.417 ± 0.244	94.1	7.15
4	4.357 ± 0.040	94.1	2.72
5	5.170 ± 0.335	91.5	3.42
Urine #2			
0	1.890 ± 0.078	Not applicable	3.44
1	2.680 ± 0.122	79.0	4.59
2	3.643 ± 0.211	87.7	5.79
3	4.597 ± 0.040	90.2	0.88
4	5.557 ± 0.033	91.7	6.03
5	6.043 ± 0.216	83.6	3.57

Urine samples #1 and #2 were analyzed in parallel and in triplicate

The *S*/*N* values were 89 in the urine of volunteer #1 and 484 in the urine of volunteer #1. Extrapolation of these *S*/*N* values to 10:1 results in limits of quantitation (LOQ) of the method of 37 nM and 67 nM Hyp, respectively. Extrapolating the *S*/*N* values to 3:1 and considering the basal Hyp concentration in the urine samples, the LOD of the GC–MS method is calculated to be 1.1 fmol and 0.56 fmol Hyp, respectively.

### Hypusine concentrations in boys’ urine of the ASOS study

**Fig. 9:**
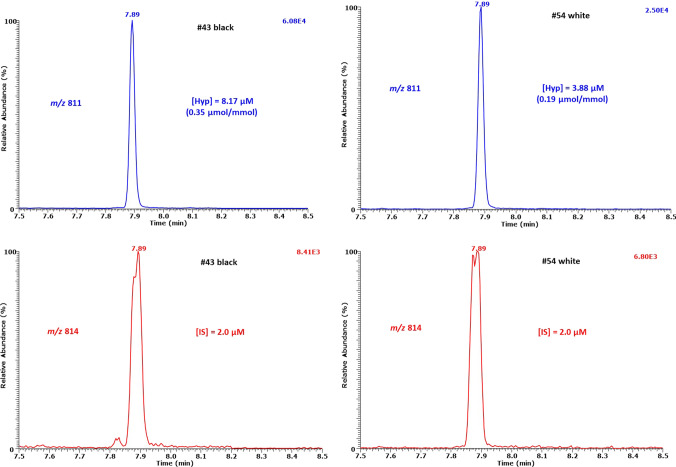
Partial chromatograms from the GC–MS analysis of endogenous hypusine in 10-µL aliquots of spot urine samples from two boys of the ASOS study. SIM of *m*/*z* 811 for endogenous Hyp and *m*/*z* 814 for the internal standard was performed. The GC oven temperature program #2 was used. The concentration of the internal standard (IS) d_3_MeHyp was each 2.0 µM in both urine samples. The concentrations and the creatinine-corrected excretion rates of endogenous hypusine in the urine samples are inserted

**Fig. 10:**
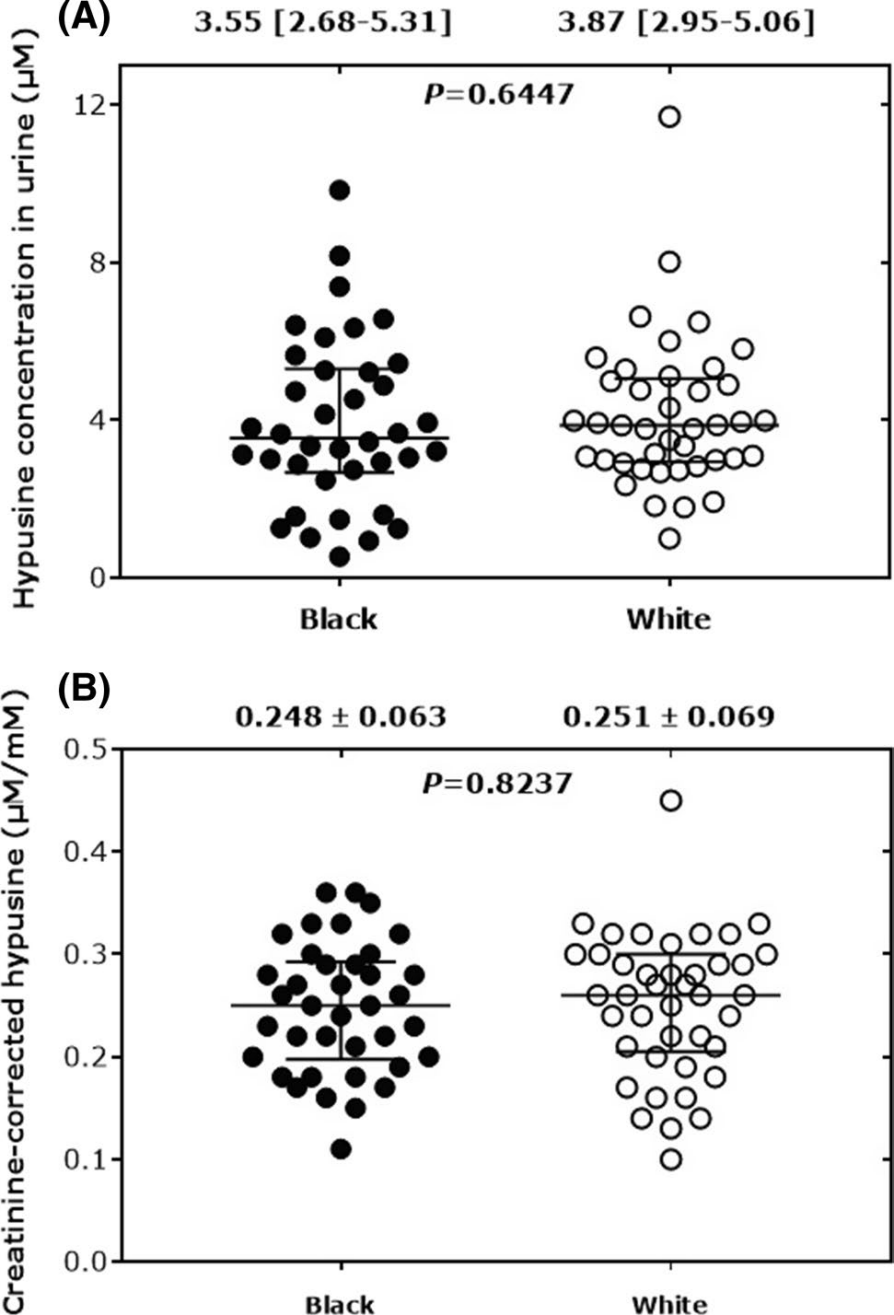
Hypusine (**A**) and creatinine-corrected excretion rate of hypusine (**B**) in the black (*n* = 38) and white (*n* = 41) children of the ASOS study. Data are shown as mean ± standard deviation (**A**) and as median with interquartile range (**B**)

The urinary creatinine concentrations did not differ between black and white boys (median with interquartile range (IQR): 15.1 [10.1–20.0] mM vs. 15.9 [12.8–18.8] mM, *P* = 0.504). Representative GC–MS chromatograms from the measurement of Hyp in two urine samples of the ASOS study are shown in Fig. 9. Endogenous Hyp was detected in all urine samples. The Hyp concentration in the urine was 3.55 [2.68–5.31] µM (range 0.54–9.84 µM) in the black boys and 3.87 [2.95–5.06] µM (range 1.0–11.7 µM) in the white boys (*P* = 0.64) (Fig. 10A). The creatinine-corrected excretion rates were 0.25 [0.20–0.29] µmol/mmol (range 0.11–0.36 µmol/mmol) in the black boys and 0.26 [0.21–0.30] µmol/mmol (range 0.10–0.45 µmol/mmol) in the white boys (*P* = 0.82) (Fig. 10B). Urinary Hyp did not correlate with the children’s age (*P* = 0.50). These results suggest that there is no ethnic-related difference in the ASOS population in the modified eIF5A protein. The Hyp concentrations and excretion rates we measured in the urine of the children of the ASOS study from South Africa are comparable to those reported for healthy children from Japan (Nakajima et al. 1971) and the USA (Woody and Pupene 1973). Urinary Hyp excretion did not depend on the boys’ age.

The correlations found between the creatinine-corrected excretions of Hyp and other analytes in the urine samples of the ASOS study are summarized in Table 2. Interestingly, we found considerable ethnic-related differences regarding correlations after Spearman’s analysis between the creatinine-corrected excretion rates of Hyp as measured in the present work, several amino acids and their AGEs (Baskal et al. 2021) and that of nitrite (Tsikas et al. 2018) as measured previously. The Hyp excretion rate did not correlate with the excretion rate of Lys. In the white boys, Hyp excretion correlated inversely with the excretion of Glu + Gln and Asp + Asn. In the black boys, Hyp excretion correlated positively with several amino acids and AGEs as listed in Table 2. The highest correlations were observed for Ala, Phe and Thr. Hyp excretion rate did not correlate with the excretion rates of nitrite, nitrate or malondialdehyde (MDA). Nitrite is a minor urinary autoxidation metabolite of nitric oxide (NO), which is derived from l-arginine by the catalytic action of NO synthase isoforms (Tsikas 2008). Nitrate is the major urinary metabolite of NO (Tsikas 2008). MDA is a biomarker of oxidative stress, notably of lipid peroxidation (Tsikas 2017b). The meaning of existing and lacking correlations between Hyp, amino acids, oxidative stress (MDA) and NO synthesis remains to be elucidated.

**Table 2:** Spearman’s coefficients of correlation (*r*) and *P* values between the creatinine-corrected excretion rates of hypusine and those of the indicated amino acids and their advanced glycation end-products

Hypusine versus	Black boys	White boys
Amino acids		
Ala	*r* = 0.5089, *P* = 0.0011	None
Phe	*r* = 0.4876, *P* = 0.001	None
Thr	*r* = 0.4873, *P* = 0.0019	None
Glu + Gln	*r* = 0.4365, *P* = 0.0061	*r* = − 0.407, *P* = 0.0083
Asp + Asn	None	*r* = − 0.3801, *P* = 0.0142
Trp	*r* = 0.3548, *P* = 0.0289	None
Orn + Cit	*r* = 0.3492, *P* = 0.0317	None
Leu + Ile	*r* = 0.3319, *P* = 0.0418	None
Advanced glycation end-products		
Carboxymethyllysine	*r* = 0.3706, *P* = 0.0220	None
Carboxymethylarginine	*r* = 0.3686, *P* = 0.0228	None
(2-Succinyl)cysteine	*r* = 0.3632, *P* = 0.0210	None

## Discussion

### GC–MS analysis of hypusine in human urine

The first scientific publication on the occurrence of hypusine (Hyp) refers to bovine brain and goes back to 1971 (Shiba et al. 1971). Hypusine is an usual putrescine–lysine derivative, *N*^ε^-(4-amino-2-hydroxybutyl)lysine, a hybrid of the proteinogenic amino acid lysine and the polyamine putrescine. According to the present knowledge, Hyp originates from a single protein, i.e., the eukaryotic initiation factor eIF5A (Park et al. 2021). Half a century ago, Hyp was identified and quantified in various mammalian organs, in human urine and in human serum by means of commercially available amino acid analyzers (Nakajima et al. 1971; Woody and Pupene 1973). Reported concentrations of Hyp in urine of healthy humans were in the lower µM-range and accounted to about 100 nmol/mmol creatinine. In serum of healthy and hyperlysinemia subjects, Hyp concentrations were reported to be below 200 nM, presumably the limit of quantitation for Hyp of these methods. The analytical performances of these approaches in terms of accuracy, precision and sensitivity have not been reported thus far. Subsequent investigations on Hyp focused mainly on the use of Hyp in biochemical and pharmacological studies. The present work deals with the development, validation and application of a stable-isotope dilution GC–MS method for Hyp based on previously reported reliable methods for amino acids, including Lys, and its metabolites of post-translational modifications (PTM), such as enzymatic *N*^ε^-methylation and chemical *N*^ε^-glycation (Hanff et al. 2019, 2020; Baskal et al. 2021). The investigations reported in the present work are based on the use of commercially available synthetic Hyp, i.e., (2*S*)-hypusine dihydrochloride. This reference compound has been declared by the manufacturer to have a chemical purity of ≥ 95% as determined by HPLC. However, no other information was provided about its synthesis and the nature of potential impurities in the Hyp preparation.

The present work indicates that the two-step derivatization procedure and the GC–MS analysis previously reported for various amino acids and their metabolites (Hanff et al. 2019; Baskal et al. 2021) are also useful for the quantitative measurement of Hyp in human urine. The derivatization and the GC–MS analysis of Hyp resemble those of 5-hydroxy-lysine (Baskal et al. 2021), especially with respect to the acylation of the hydroxy groups with PFPA. The methyl ester of d,l-5-hydroxy-l-lysine reacts with PFPA to form *N*^α^,*N*^ε^,*O*^5^-(PFP)_3_, indicating acylation of the 5-hydroxy group (Baskal et al. 2021). Yet, there is a difference between these amino acids. The GC–MS spectrum of the Me-PFP derivative of d,l-5-hydroxy-l-lysine contains the mass fragment *m*/*z* 163 as the base peak (intensity, 100%) due to the formation of penta-fluoro-propionate (CF_3_CF_2_COO^−^) (Baskal et al. 2021). The mass fragment *m*/*z* 163 is also present in the mass spectra of the peaks eluting at 11.9 min, but it is very weak (Fig. 2). This suggests that the hydroxy group of Hyp is acylated, but ionizes differently than the acylated hydroxy group of the Me-PFP derivatives of d,l-5-hydroxy-l-lysine. The mass fragment *m*/*z* 163 is also present in the mass spectra of the peaks eluting at 12.3 min at much lower intensity (Fig. 3), suggesting that the acylated hydroxy group of these derivatives ionizes differently than the acylated hydroxy group of the Me-PFP derivatives of d,l-5-hydroxy-l-lysine. The largest GC–MS peak of Hyp is likely to be Hyp-d_0_Me-(*N,N,N,N,O*-PFP)_5_, i.e., an entirely derivatized Hyp. The second large GC–MS peak of derivatized Hyp seems to be Hyp-d_0_Me-(*N,N,N,N*-PFP)_4_, i.e., to have a native non-derivatized hydroxyl group. The other two GC–MS peaks of derivatized Hyp are relatively small. Yet, the present study cannot state whether they originate from impurities present in the commercially available preparation (2*S*)-hypusine dihydrochloride. There is some indication that the lastly eluting GC–MS peak could be the Me-PFP derivative of deoxyhypusine (dHyp-d_0_Me-(*N,N,N,N*-PFP)_4_). However, we could not clarify this issue because currently, deoxy-hypusine is not commercially available. The Me-PFP derivatives of the hydroxylated amino acids of Lys and Pro, i.e., d,l-5-hydroxy-l-lysine and 4-hydroxy-proline, elute in front of the Me-PFP derivatives of their precursors. The relative retention time of the Me-PFP derivatives of dHyp and Hyp is 1.1144, which is close to that of Lys and Pro with respect to d,l-5-hydroxy-l-lysine: 1.1088 and 1.0512, respectively (using GC oven temperature program #1). This indicates that the acylation of hydroxy groups of amino acids with PFPA decreases considerably their retention time in the GC column, although the derivatives are larger. The relative retention time of Hyp to dHyp in GC–MS is about 1.0337 (von Koschitzky et al. 2015), suggesting that etherification of the hydroxyl group Hyp also increases the volatility of the analyte.

The very similar total ion current values of the chromatograms (Fig. 1) and of the mass spectra (Figs. 2, 3) of derivatized Hyp suggest that the two-step derivatization procedure and the NICI process are well reproducible and both derivatives of Hyp are very thermally stable and should be useful in quantitative analyses. We focus on the ion *m*/*z* 811 of the Hyp derivative Hyp-d_0_Me-(*N,N,N,N,O*-PFP)_5_. The ion *m*/*z* 811 is much higher than the *m*/*z* values of all other ions we found for Me-PFP derivatives of the amino acids (Hanff et al. 2019; Baskal et al. 2021) and even of the tripeptide glutathione (Bollenbach and Tsikas 2020). The *m*/*z* value of 811 is also larger than those of PFP derivatives of polyamines including spermidine and putrescine (Hanff et al. 2020). The GC–MS method reported herein may be highly specific for Hyp in human urine. We presume that this GC–MS method would also be useful to measure deoxyhypusine*, N*^ε^-(4-amino-butyl)lysine, the Me-PFP derivative of which is expected to elute behind the Hyp peak and to have *m/z* 649 as the most intense mass fragment in the NICI mode.

### Physiological occurrence of hypusine in human urine

The GC–MS method presented in this work is accurate, precise and sensitive enough to measure Hyp in human urine. Our study shows that Hyp is physiologically present in the urine of two healthy young subjects at concentrations of 0.6 µM and 1.9 µM corresponding to about 0.29 µmol/mmol creatinine and 0.19 µmol Hyp/mmol creatinine in these subjects. In urine samples of black and white children from South Africa, the GC–MS methods provided Hyp concentrations and excretion rates of the same order of magnitude as in adults. Such Hyp concentrations in the urine of healthy and ill subjects including children have been reported by only two groups thus far using automated amino acid analyzers (Nakajima et al. 1971; Woody and Pupene 1973). It is worth mentioning that the occurrence of deoxyhypusine in human urine has not been reported previously (Nakajima et al. 1971; Woody and Pupene 1973). In in vitro, both Hyp and dHyp were detected with purified DHPS and DOHH (Kaiser et al. 2012). Given the high reliability, the GC–MS method reported here should be useful for the quantitative determination of Hyp in other relevant biological samples, such as human serum and plasma. In the urine samples of the two volunteers analyzed in the present study, we did not find deoxyhypusine, assuming that deoxyhypusine forms a derivative dHyp-d_0_Me-(*N,N,N,N*-PFP)_4_ which ionizes to *m*/*z* 650 under NICI conditions.

The Hyp concentrations we measured in the urine of the children of the ASOS study from South Africa are comparable to those reported for healthy children of comparable age from Japan (Nakajima et al. 1971) and the USA (Woody and Pupene 1973). The comparability of the urinary Hyp concentrations may suggest that the eIF5A hypusination is ethnic-independent. Nevertheless, the differences found between the correlations of many urinary amino acids including their AGEs and urinary Hyp in the children of the ASOS study suggest that not yet known differences in amino acid homoeostasis including metabolism to polyamine and excretion may depend upon ethnicity. Spermidine is the biological precursor of Hyp (Park et al. 1981, 2021). At high mM concentrations, spermidine was reported to enhance weakly hypusination in flies and mice (Liang et al. 2021). In the ASOS study, children did not receive supplementary amino acids or spermidine. Previously, urinary metabolomics investigations in the ASOS study revealed a link with premature arterial stiffness in black boys (Erasmus et al. 2019). It remains to be investigated whether such a link also exists for Hyp.

### Occurrence of hypusine and hypusinated eIF5A in human plasma and serum

Until the present day, eIF5A is considered the only protein that is hypusinated (Scheme 1). On the other hand, there are numerous proteins that are modified by other more general and abundant PTM. Nevertheless, the concentration of free biomarkers that are released by proteolysis of such proteins, circulate in blood and are excreted in the urine, is of the order of Hyp or even lower. This raises the question, whether the biosynthetic turnover of eIF5A/eIF5A^HYP^ is much higher than presently known.

In rat plasma, the concentration of eIF5A including its hypusinated (on Lys) and sulphated (on Tyr) forms was measured by a sandwich ELISA to be around 16 ng/mL corresponding to about 1 nM (Yao et al. 2016a). In plasma of healthy subjects, the concentration of eIF5A including its hypusinated and sulphated forms was measured by a sandwich ELISA to be around 9.9 ng/mL (Yao et al. 2016b). Using the same ELISA assay, this group reported plasma eIF5A concentrations of patients with familial cholesterolemia considerably higher than in plasma of control subjects (about 150 ng/mL vs. 10 ng/mL) (Sato et al. 2021). Such very low concentrations suggest that measurement of hypusinated eIF5A in human plasma would be not successful by the GC–MS method reported in the present article.

## Abbreviations

AGEs: Advanced glycation end-products
ASOS: Arterial Stiffness in Offspring Study
DHPS: Deoxyhypusine synthase
dHyp: Deoxyhypusine
DOHH: Deoxyhypusine hydrolase
EA: Ethyl acetate
EI: Electron ionization
eIF5A: Eukaryotic initiation factor 5A
GC–MS: Gas chromatography–mass spectrometry
HPLC: High-performance liquid chromatography
Hyp: Hypusine
IQR: Interquartile range
IS: Internal standard
LOD: Limit of detection
LOQ: Limit of quantification
MM: Molecular mass
MDA: Malondialdehyde
Me: Methyl; methyl ester
*m/z*: Mass-to-charge ratio
NICI: Negative-ion chemical ionization
NO: Nitric oxide
PAR: Peak area ratio
PFB-Br: Pentafluorobenzyl bromide
PFP: Penta-fluoro-propionyl
PFPA: Penta-fluoro-propionic anhydride
PFPOH: Penta-fluoro-propionic acid
PTM: Post-translational modification
SIM: Selected-ion monitoring
*S*/*N*: Signal-to-noise ratio
UPLC: Ultra-performance liquid chromatography
